# Dapsone protects brain microvascular integrity from high-fat diet induced LDL oxidation

**DOI:** 10.1038/s41419-018-0739-y

**Published:** 2018-06-07

**Authors:** Rui Zhan, Mingming Zhao, Ting Zhou, Yue Chen, Weiwei Yu, Lei Zhao, Tao Zhang, Hecheng Wang, Huan Yang, Yinglan Jin, Qihua He, Xiaoda Yang, Xiangyang Guo, Belinda Willard, Bing Pan, Yining Huang, Yingyu Chen, Dehua Chui, Lemin Zheng

**Affiliations:** 1 Neuroscience Research Institute and Department of Neurobiology, Key Laboratory for Neuroscience, Ministry of Education and Ministry of Public Health, Health Science Center, Peking University, Beijing, China; 2 The Institute of Cardiovascular Sciences and Institute of Systems Biomedicine, School of Basic Medical Sciences, Key Laboratory of Molecular Cardiovascular Science, Ministry of Education, Key Laboratory of Cardiovascular Molecular Biology and Regulatory Peptides, Ministry of Health, Beijing Key Laboratory of Cardiovascular Receptors Research, Peking University Health Science Center, 100191 Beijing, China; 3 Department of Neurology, The Institute of Small Vessel Disease of the Nervous System, Peking University First Hospital, Beijing, China; 4 Center of Medical and Health Analysis, Peking University, Beijing, China; 5 School of pharmaceutical sciences, Health Science Center, Peking University, Beijing, China; 6 Department of Anesthesiology, Peking University Third Hospital, Beijing, China; 7 Proteomics Laboratory, Cleveland Clinic, Cleveland, Ohio USA; 8 Department of Immunology, School of Basic Medical Sciences, Key Laboratory of Medical Immunology, Ministry of Health, Health Science Center, Peking University, Beijing, China; 9 Beijing Key Laboratory of Magnetic Resonance Imaging Devices and Technology, Peking University Third Hospital, Beijing, China

## Abstract

Atherosclerosis was considered to induce many vascular-related complications, such as acute myocardial infarction and stroke. Abnormal lipid metabolism and its peroxidation inducing blood–brain barrier (BBB) leakage were associated with the pre-clinical stage of stroke. Dapsone (DDS), an anti-inflammation and anti-oxidation drug, has been found to have protective effects on vascular. However, whether DDS has a protective role on brain microvessels during lipid oxidation had yet to be elucidated. We investigated brain microvascular integrity in a high-fat diet (HFD) mouse model. We designed this study to explore whether DDS had protective effects on brain microvessels under lipid oxidation and tried to explain the underlying mechanism. In our live optical study, we found that DDS significantly attenuated brain microvascular leakage through reducing serum oxidized low-density lipoprotein (oxLDL) in HFD mice (*p* < 0.001), and DDS significantly inhibited LDL oxidation in vitro (*p* < 0.001). Our study showed that DDS protected tight junction proteins: ZO-1 (*p* < 0.001), occludin (*p* < 0.01), claudin-5 (*p* < 0.05) of microvascular endothelial cells in vivo and in vitro. DDS reversed LAMP1 aggregation in cytoplasm, and decreased the destruction of tight junction protein: ZO-1 in vitro. We first revealed that DDS had a protective role on cerebral microvessels through preventing tight junction ZO-1 from abnormal degradation by autophagy and reducing lysosome accumulation. Our findings suggested the significance of DDS in protecting brain microvessels under lipid metabolic disorders, which revealed a novel potential therapeutic strategy in brain microvascular-related diseases.

## Introduction

Atherosclerosis is understood to be a disease characterized by inflammation and oxidative stress that results in many vascular-related complications, including ischemia, acute coronary syndromes (e.g., myocardial infarction), and stroke^1^. Lipid metabolism is regarded as a key factor in many vascular-related diseases. Lipid peroxidation, especially low-density lipoprotein (LDL) oxidation is considered a key risk factor of atherosclerosis. Under pathological conditions, oxidized LDL (oxLDL), the oxidative modified form of LDL, leads to lysosomal accumulation^2,3^. In arterial atherosclerotic plaques, significantly high level of oxLDL is observed^4^, which can induce leukocytes to release inflammatory factors. It leads to dysfunction and apoptosis of vascular endothelial cells. Therefore, oxLDL plays a central role in the pathogenesis of atherosclerosis and other vascular-related diseases^5–9^. Previous studies reported that abnormal lipid metabolism exacerbated blood–brain barrier (BBB) impairment in mice with brain injury^10,11^. BBB maintenance is important in the central nervous system (CNS) because disruption of the BBB may contribute to many brain disorders, including Alzheimer’s disease and ischemic stroke^12^.

Autophagy is a lysosome-dependent degradation pathway involved in the pathogenesis of many human diseases, especially cancer, neurodegeneration disease and cardiovascular disease^13,14^. Many previous evidence revealed that the vascular function played a key role in the progression of Alzheimer’s disease, Parkinson’s disease and atherosclerosis^15,16^. The precise role of autophagosome–lysosome in regulating vascular endothelium function in vivo remains to be elucidated.

Dapsone (DDS) has traditionally been widely used in dermatosis therapy^17^. In recent studies, DDS has been shown to play an anti-aging role, which is discovered by extending organismic lifespan in the *Caenorhabditis elegans* model^18^. Other studies reported that DDS has also exhibited neuroprotective effects in stroke patients^19^. DDS has been shown to prevent neuron injury in the hippocampus of Alzheimer’s disease patients^20^. DDS is a treatment for primary immune thrombocytopenia (ITP), with response rates of 27% to 63%^21^. Moreover, DDS has been shown to accelerate wound healing in diabetes mellitus^22^. Whereas the underlying mechanisms have not been elucidated. Our previous study reported that DDS suppressed surgical stress-induced cognitive impairment through its anti-oxidation effects and preventing vascular injury from inflammation^23–25^. However, whether DDS protects brain microvascular integrity through regulating autophagy and lysosome functions is still unknown.

Previous studies reported that DDS had anti-oxidative effects on reactive oxygen species (ROS)^26^. And in the study of DDS extended organismic lifespan in the *Caenorhabditis elegans*, the authors reported that the lifespan delaying was because of DDS targeted to pyruvate kinase (PK) and decreased the level of a mitochondrial complex^18^. Many studies have been shown that the western diets were related to our organism aging, metabolic syndromes and perhaps owed to vascular endothelial dysfunction^10,11^. So, we hypothesized that whether DDS influences vascular endothelial function through its anti-oxidative effect under the environmental risk factors of abnormal lipid oxidation and chronic inflammation. In this study, we demonstrate that DDS protected brain microvascular integrity via its anti-oxidative role and reducing lysosome accumulation in a high-fat diet (HFD) mouse model that mimic human western diets, which indicate that DDS can design to be a novel therapeutic candidate in brain vascular-related disorders under metabolism disorders, such as stroke and Alzheimer’s disease.

## Results

### Result 1: DDS protects brain microvascular integrity in mice with HFD

**Fig. 1:**
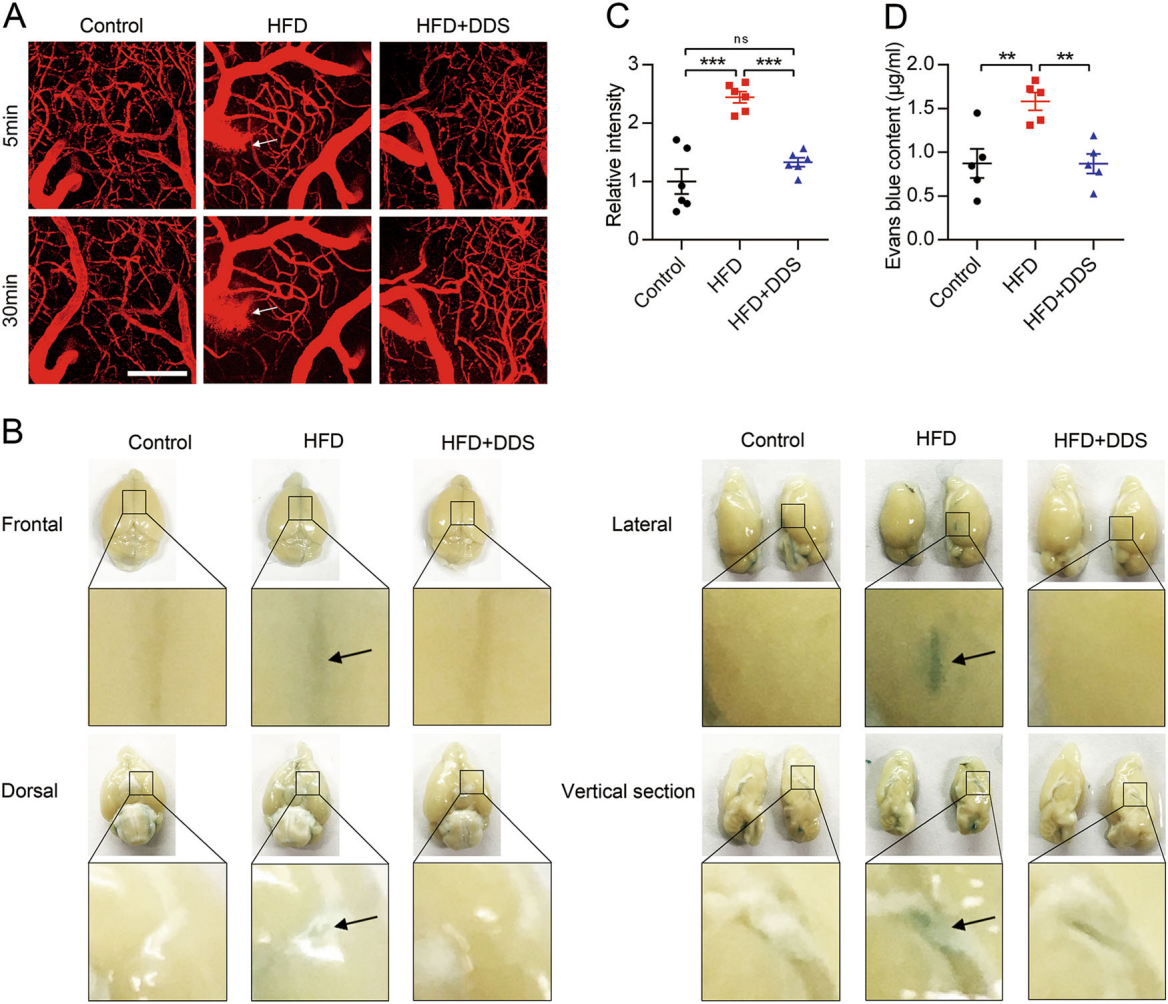
DDS prevents brain microvessels integrity from impairment during HFD. **a** Live optical multiphoton laser confocal microscopy of TMR-Dextran (red) was used to observe brain microvascular integrity in control, HFD and HFD + DDS mice. Scale bar, 100 μm. **b** The extravasation of Evans blue dye into brain parenchyma on the front, behind, lateral and longitudinal section of mice brain. **c** Quantification of the relative fluorescence intensity across cross-section of vessels was calculated from each group in Fig. 1a. Mean ± SEM. *n* = 6 animals per group. **d** Quantification of the amount of Evans blue dye extravasation in the parenchyma of mice brain. Data were represented as mean ± SEM, *n* = 5 animals per group. The groups were compared by one-way ANOVA and followed by Dunnett’s multiple comparison tests. Differences were considered significant at **p < 0.01, ***p < 0.001

To determine whether DDS has protective effects on brain microvessels, we examined the cortical microvascular permeability for tetramethylrhodamine (TMR)-dextran (40 kDa) with multiphoton microscopy in a live optical study. We showed intact microvessel networks in control mice. There are leaky microvessels (as arrows show, dye leaks come with time) in HFD mice (Fig. 1a). The relative fluorescence intensity across out vessels from HFD mice was 2.4-fold higher than control (Fig. 1c, *F* = 28.55, *R*^2^ = 0.7920, *p* < 0.0001, *n* = 6). Administration of DDS significantly attenuated HFD-induced microvessel leakage (Fig. 1a), and the relative fluorescence intensity decreased (Fig. 1c, *p* = 0.0001, *n* = 6). There was no significant difference between control and HFD + DDS group (*p* = 0.2039).

The extravasation of Evans blue dye from brain vessels into brain parenchyma is a sign of brain microvascular integrity^27^. To confirm our primary findings, we investigated the Evans blue into the brain in HFD mice models. There was little extravasation of dye into the brain in control mice. However, we found that HFD significantly induced leakage of microvessels, which was significantly attenuated by DDS (Fig. 1b). The amount of Evans blue in brain parenchyma was detected in the following experiment. Consistently, the content of dye into brain from HFD + DDS group was lower than HFD group (Fig. 1d, *F* = 9.964, *R*^2^ = 0.6242, *p* = 0.041, *n* = 5). These data indicated that DDS protects brain microvascular integrity during HFD in vivo.

### Result 2: DDS reduces the level of serum oxLDL level in vivo, and has anti-oxidation effects on oxLDL in vitro

**Fig. 2:**
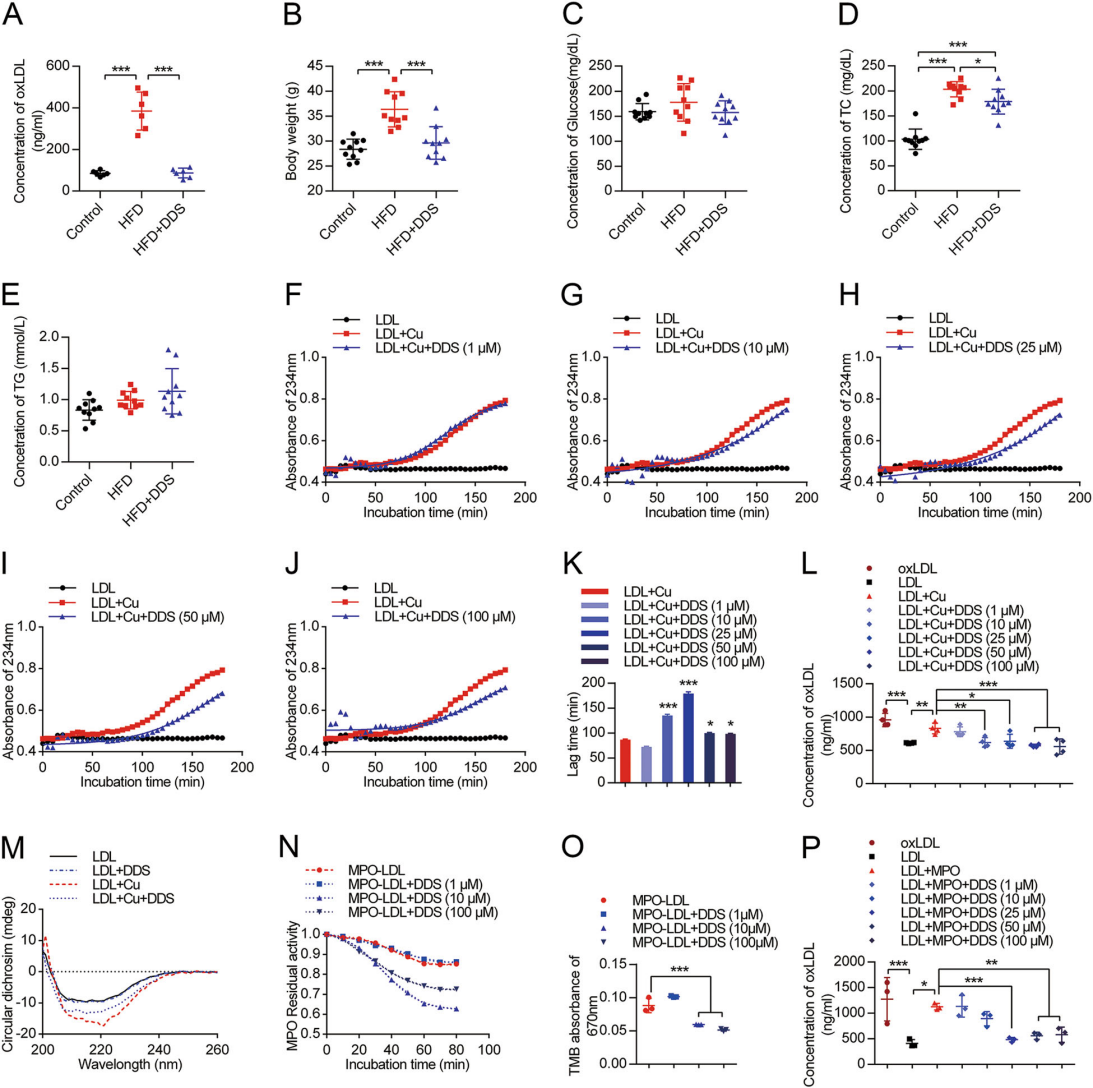
DDS decreases the level of oxLDL. **a** Blood serum oxLDL content was detected by oxLDL ELISA kit in control, HFD and DDS treated HFD groups. **b** Body weight, **c** serum glucose, **d** TC, **e** TG were measured in previous groups. The results were represented as mean ± SEM, *n* = 6–10 animals per group. The groups were compared by one-way ANOVA and followed by Dunnett’s multiple comparison tests. Differences were considered significant at ***p < 0.001. **f-j** Measurement of the kinetics of oxidation of LDL by continuously monitoring the change of UV absorbance at 234 nm; the UV absorbance curve of different groups: native LDL treatment with BHT, LDL-oxidized modification by Cu^2+^ and the treatment with dose-dependent DDS (1 µM, 10 µM, 25 µM, 50 µM, 100 µM). These three treatments were mimicked by the Boltzman sigmoidal nonlinear regression model. **k** The lag time of LDL oxidation in each groups. **l** oxLDL content by an oxLDL ELISA kit. The results were represented as mean ± SEM of four independent experiments. The treatment groups were compared by one-way ANOVA and followed by Dunnett’s multiple comparison tests. Differences were considered significant at *p < 0.05, **p < 0.01, ***p < 0.001. **m** CD measurement of control group, LDL treated with Cu^2+^ group, LDL oxidized with Cu^2+^ pre-treatment with DDS group. **n** MPO residual activity detected with absorbance at 670 nm. **o** The statistical analysis of MPO peroxidase activity. **p** oxLDL was detected using an ELISA kit. Mean ± SEM of three independent experiments. The treatment groups were compared by one-way ANOVA and followed by Dunnett’s multiple comparison tests. Differences were considered significant at *p < 0.05, **p < 0.01, ***p < 0.001

Next, we assessed the influence of DDS on the level of serum oxLDL, body weight, the level of serum glucose, total cholesterol (TC) and triacylglyceride (TG) in mice. Compared with the control, we observed that HFD mice had a higher oxLDL level. Interestingly, DDS treatment significantly decreased the oxLDL level (Fig. 2a, *F* = 58.76, *R*^2^ = 0.8868, *p* < 0.0001, *n* = 6). Moreover, DDS also significantly decreased the body weight of HFD mice (Fig. 2b). There were no significant differences in serum glucose level, and TG among these groups (Fig. 2c-e). There was significant difference of TC level between control and HFD group (*p* < 0.0001). In HFD + DDS group, DDS significantly decreased TC level compared with HFD group (*p* = 0.0229). And there was significant difference of TC level between control and HFD + DDS group (*p* < 0.0001). These results indicated that DDS decreased the level of serum oxLDL in HFD mice.

In the following study, we investigated whether DDS could show anti-oxidative effects on the oxidation of LDL in vitro. We designed the process of Cu^2+^ as an oxidant to oxidize LDL. The experiments showed that DDS inhibited oxLDL generation. Some reports attributed the suppression of oxygen-radical generation ability to DDS. In the current study, we supposed that DDS had the capacity to inhibit oxidation by Cu^2+^. We found an increasing time lag in Cu^2+^ oxidized LDL with median dose DDS treatment, 25 μM DDS treatment was the most suitable concentration for increasing lag time (*p* < 0.0001, for 50 μM DDS here, *p* = 0.0186), 50 μM DDS treatment was the most suitable concentration for anti-oxidation of the LDL (*p* = 0.0008, for 25 μM DDS here, *p* = 0.0137), which suggested that DDS had significant anti-oxidation effect in vitro (Fig. 2f-l). We further wondered whether DDS directly bound to LDL, hindered Cu^2+^ from interacting with LDL, and suppressed the oxidation process. We used circular dichroism (CD) spectrum to observe the change of the structure during LDL treatment with DDS, and the opposite change after LDL oxidation (Fig. 2m). These results suggested that DDS exerted its anti-oxidative effect through directly binding to the protein portion of LDL, ApoB-100. This finding will help explain the mechanism by which DDS directly interacts with LDL to inhibit LDL from oxidation.

**Fig. 3:**
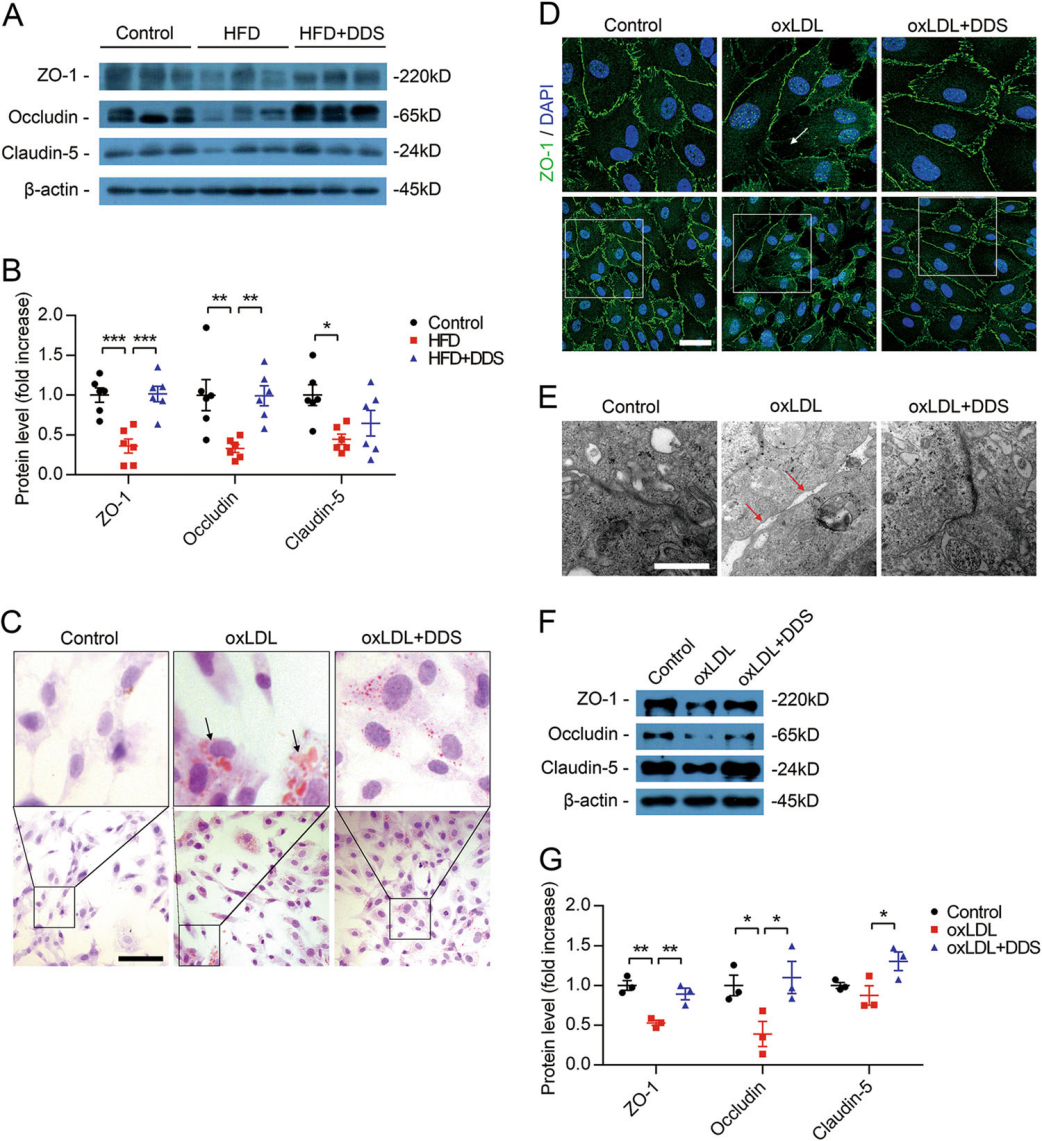
DDS preserves tight junctions and decreases intracellular oxLDL. **a** Immunoblotting for tight junction proteins: ZO-1, occludin, and claudin-5 in isolated brain microvessels were assessed in control, HFD and DDS treated HFD mice. **b** Quantitative analysis of ZO-1, occludin, and claudin-5. β-Actin was used as control. The results were represented as mean ± SEM, *n* = 6 animals per group. **c** Oil red O staining for intracellular oxLDL in HBMECs. Scale bar, 100 μm. **d** Confocal microscopy for tight junction protein ZO-1 (green), nucleus (blue, stained with DAPI) in control, oxLDL, and DDS treated oxLDL groups. Scale bar, 50 μm. **e** TEM analysis for tight junctions in control, oxLDL, and DDS treated oxLDL groups. Scale bar, 1 μm. **f** Immunoblotting for tight junction proteins: ZO-1, occludin, and claudin-5 were assessed in control, oxLDL and DDS-treated oxLDL groups. **g** Quantitative analysis of ZO-1, occludin, and claudin-5. β-Actin was used as control. The results were represented as mean ± SEM, *n* = 3 independent experiments per group. The groups were compared by one-way ANOVA and followed by Dunnett’s multiple comparison tests. Differences were considered significant at *p < 0.05, **p < 0.01, ***p < 0.001

The highly oxidized and aggregated LDL in the vessel, in many ways, was a result of ROS. These ROS included divalent copper ion, heme, and different enzyme systems, such as myeloperoxidase (MPO), lipoxygenases, and NADPH oxidases^28,29^. In this process, MPO plays a key role in the oxidation and aggregation of LDL in the arterial wall^30,31^. Not only the Cu^2+^ but also the MPO-H_2_O_2_-Cl^−^ system can be used in vitro to elucidate the potential mechanisms of LDL oxidation^32^. Thus, in our following experiments, we mimicked the process of LDL oxidation by the MPO-H_2_O_2_-Cl^−^ system. An interesting result was discovered: DDS had an anti-oxidative effect, and inhibited oxLDL generation (Figs. 2n-p). These results indicated that DDS played an anti-oxidative role and inhibited MPO peroxidase activity.

### Result 3: DDS protects tight junction proteins and reduces intracellular oxLDL in vivo and in vitro

Brain microvascular integrity is maintained by the presence of tight junction proteins. Disruption of the tight junctions of the BBB is a hallmark of many pathologies, including stroke, HIV encephalitis, Alzheimer’s disease and so on^33^. We used immunoblotting to assess the protein levels of the tight junction proteins: occludin, claudin-5 and tight junction-associated proteins Zonula occludens-1 (ZO-1) in the brain vessels of the mice. The results showed that HFD decreased the levels of ZO-1, occludin and claudin-5 compared with control, respectively (Fig. 3a, b). Compared with the HFD group, DDS restored the levels of tight junction proteins: ZO-1 (*p* = 0.0003), occludin (*p* = 0.0069), but not claudin-5 (*p* = 0.4373) (Fig. 3a, b). These results indicated that DDS protects tight junction proteins, which were decreased during HFD.

To understand the underlying mechanism of how DDS protect tight junction proteins, we used oxLDL to treat endothelial cells, and we confirmed our previous findings in vivo. We detected intracellular oxLDL by oil red O staining^34^, and we found that DDS reduced the level of intracellular oxLDL in human brain microvessel endothelial cells (HBMECs) during oxLDL treatment (Fig. 3c).

Following, we observed tight junction disruption induced by oxLDL could be alleviated by DDS (Fig. 3d). These results were consistent with the observation from transmission electron microscope (TEM) (Fig. 3e) and the immunoblotting in cells (Fig. 3f). We also found abnormal cell morphology (spindle shape) treated with 200 μg/ml oxLDL, which was not observed in the 200 μg/ml LDL-treated group. Compared with the oxLDL group, DDS recovered cell morphology partially to the control group (Supplementary Fig. 1A). The results showed that 200 μg/ml and 24 h were the optimal conditions for oxLDL treatment, and 25 nM and 24 h were the optimal conditions for DDS treatment (Supplementary Fig. 1B). These results indicated that DDS also alleviated oxLDL-induced tight junction destruction in vitro.

### Result 4: DDS protects tight junction through rescuing lysosome dysfunction and abnormal protein degradation

**Fig. 4:**
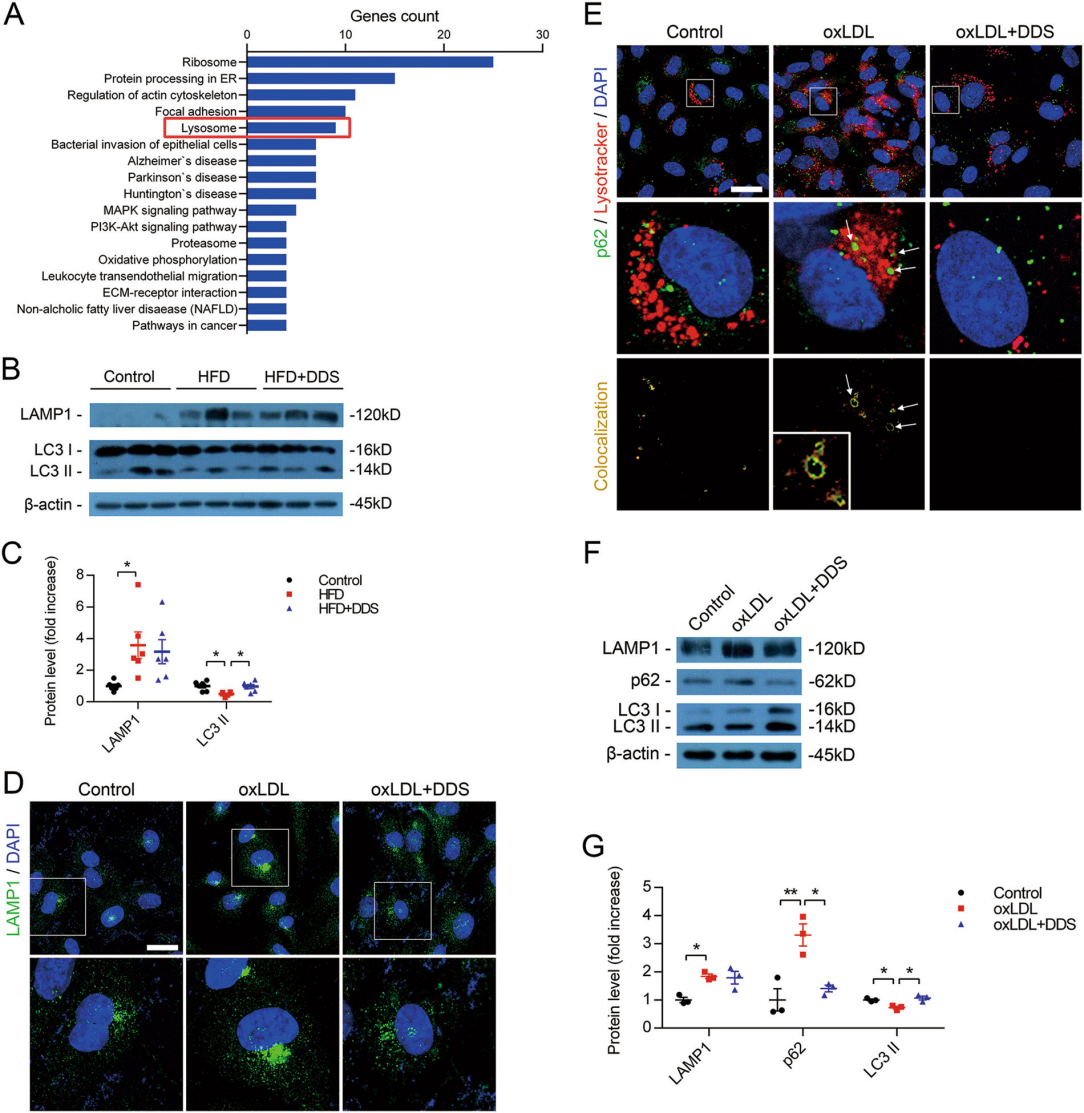
DDS attenuates lysosome dysfunction and inhibits abnormal degradation. **a** KEGG pathway analysis of changed proteins among control, oxLDL and oxLDL + DDS treatment groups. **b** Immunoblotting for autophagy-lysosome related proteins: LAMP1, LC-3 in isolated brain microvessels were assessed in control, HFD and DDS treated HFD mice, respectively. **c** Quantitative analysis of LAMP1 and LC-3. β-Actin was used as control. Mean ± SEM, *n* = 6 animals per group. **d** Confocal microscopy for lysosome membrane marker LAMP1 (green) in control, oxLDL and DDS treated oxLDL groups. **e** Autophagy substrates protein p62 (green) colocalized with lysosomes (red, stained by Lysotracker). Scale bar, 25 μm. **f** Immunoblotting for autophagy-lysosome related proteins: LAMP1, p62, and LC-3 was assessed in control, oxLDL and DDS-treated oxLDL groups. **g** Quantitative analysis of LAMP1, p62, and LC-3. β-Actin was used as control. Mean ± SEM, *n* = 3 independent experiments per group. The groups were compared by one-way ANOVA and followed by Dunnett’s multiple comparison tests. Differences were considered significant at *p < 0.05, **p < 0.01, ***p < 0.001

Using proteomic analysis, we discovered that lysosome function was significantly involved in this process (Fig. 4a and Supplementary Fig. 2A-D) (These were common pathways, such as ribosome and protein processing in ER which were found in proteomics analysis. Additionally, focal adhesion had been reported in wound healing in diabetes mellitus^22^). Previous study reported that the reduction of tight junction proteins might be related to the autophagy and lysosome degradation system^35^. Therefore, we used immunoblotting to assess the expression of autophagy-lysosome related proteins including LAMP1, and LC-3 in the brain vessels of the mice. Compared with the control, the results showed that HFD significantly downregulated the level of LC-3 II. Meanwhile, the level of LAMP1 was upregulated (Fig. 4b, c). Compared with the HFD group, DDS restored the level of LC-3 II (Fig. 4b). Taking these results together, it suggested that the protective role of DDS on tight junctions might be associated with autophagy and lysosome during HFD.

In the following investigations, we observed that oxLDL induced LAMP1 accumulation, a dysfunctional form of lysosome (Fig. 4d). We found autophagy ubiquitylation substrate p62 colocalization with lysosomes, and we observed annular colocalizations (shown by arrows), which suggested that oxLDL induced abnormal degradation of proteins. Interestingly, DDS could reverse this phenomenon (Fig. 4e). We assayed protein level by immunoblotting in cells (Fig. 4f, g) and found that the trend was consistent with the results from previous experiments (Fig. 4b). These results indicated that oxLDL induced lysosome dysfunction and abnormal protein degradation, whereas DDS attenuated lysosome dysfunction during this process.

### Result 5: DDS attenuates lysosome accumulation and activates autophagy

**Fig. 5:**
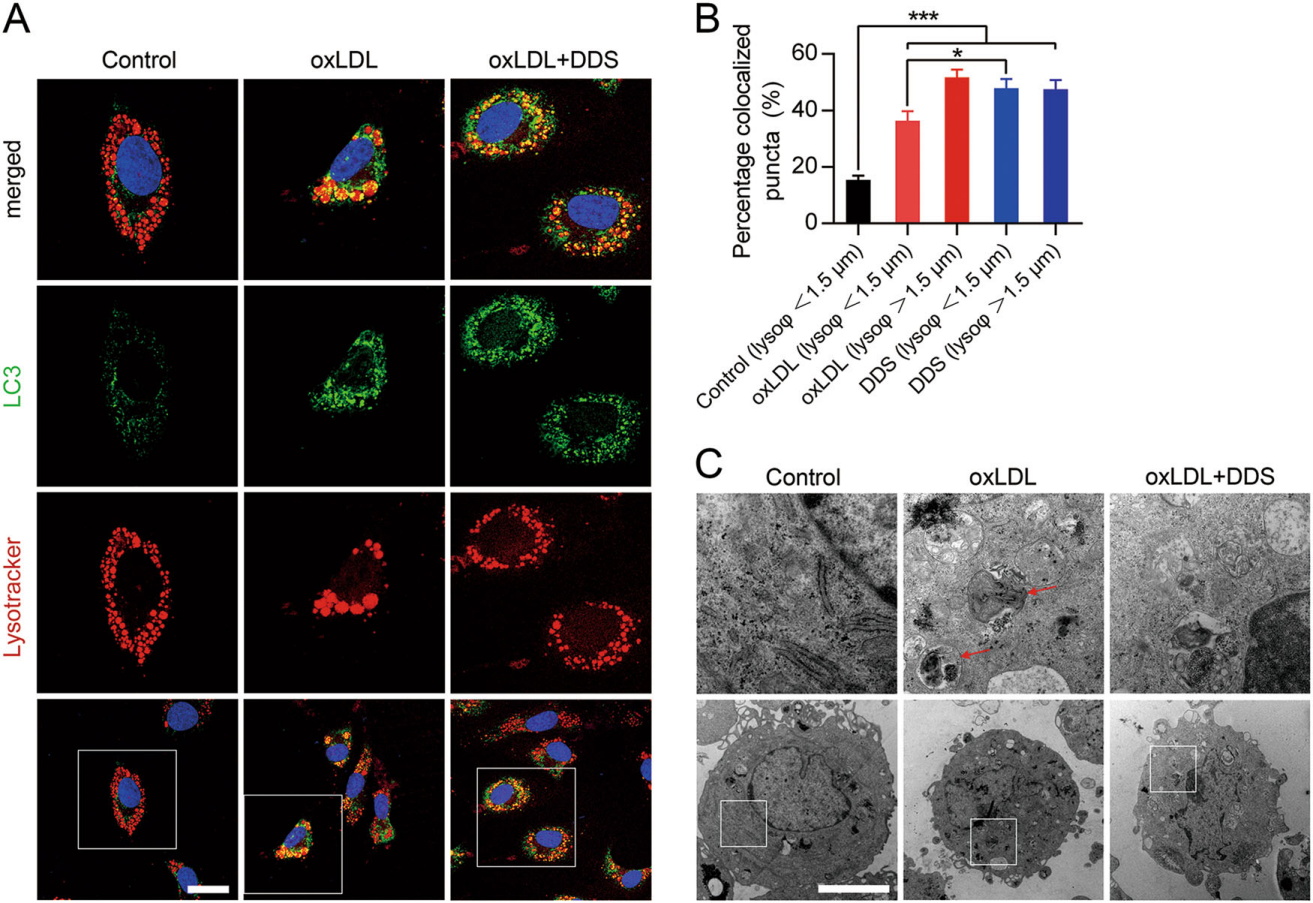
DDS depresses lysosome accumulation and accelerates autophagy. **a** Confocal microscopy for LC-3 (green) colocalization with lysosomes (red, stained by Lysotracker). Scale bar, 25 μm. **b** Quantification of LC-3 puncta colocalized with Lysotracker. Lysosomes with different diameters (normal lysosome, Φ < 1.5 μm; and accumulated lysosome, Φ > 1.5 μm) were analyzed respectively. The results were represented as mean ± SEM, *n* = 3 independent experiments per group. The groups were compared by one-way ANOVA and followed by Dunnett’s multiple comparison tests. Differences were considered significant at *p < 0.05, ***p < 0.001. **c** TEM analysis for lysosome accumulation (arrow). Scale bar, 1 μm

To confirm that DDS rescued lysosome dysfunction induced by oxLDL, we observed the colocalization of lysosomes (using Lysotracker marked) with LC-3. We found almost all of the lysosome bubble diameters were <1.5 μm in the control group, but up to 61.81% lysosomes in oxLDL-treated cells had diameters larger than 1.5 μm (counted from 55 cells). This finding indicated that oxLDL induced lysosome accumulation^36,37^. LC-3 (a widely used marker for autophagy membrane formation^38^) was colocalized with accumulated lysosomes in oxLDL treatment group. Interestingly, DDS decreased the amounts of larger-diameter lysosomes (34 cells of 55 cells in oxLDL group vs 17 cells of 57 cells in oxLDL + DDS group). DDS treatment increased the colocalization rate of LC-3 with smaller-diameter lysosomes (*F* = 37.69, *R*^2^ = 0.5258, *p* = 0.0179), which indicated that DDS activated autophagy and attenuated lysosome accumulation (Fig. 5a, b). Considering these results and the results shown in Fig. 4, we postulated that DDS protects tight junction proteins by activating autophagy and reversing lysosome accumulation.

Next, TEM was employed to observe lysosome accumulation. Consistent with our previous experiments, increased amounts of lysosome accumulation^39^ were observed in the oxLDL treatment group, whereas DDS treatment reduced lysosome accumulation (Fig. 5c).

### Result 6: DDS preserves tight junctions through decreasing ZO-1 degradation caused by abnormal autophagy

**Fig. 6:**
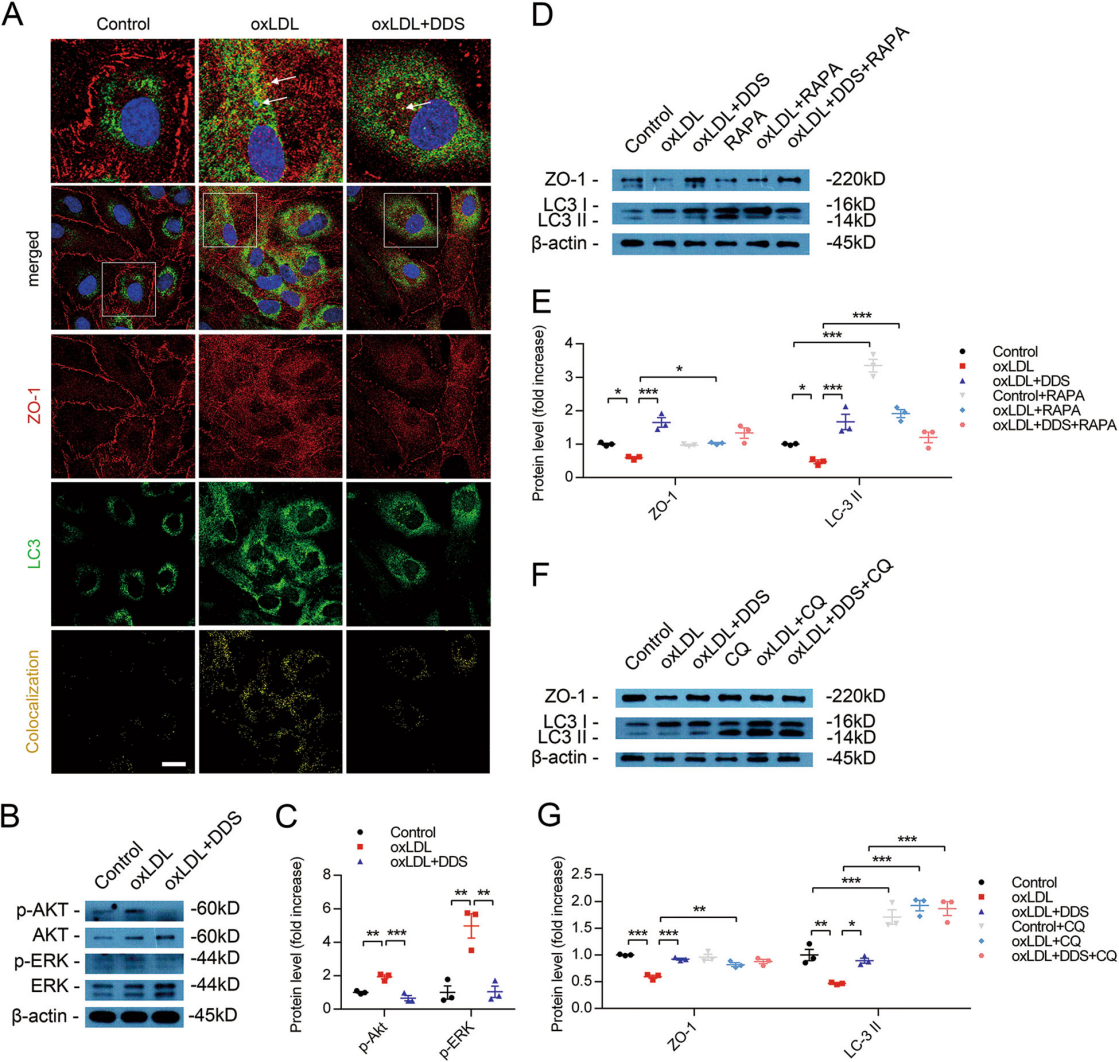
DDS prevents tight junction protein ZO-1 from abnormal degradation by autophagy. **a** Confocal microscopy for LC-3 (green) colocalization with ZO-1 (red). Scale bar, 25 μm. **b** Immunoblotting for p-Akt/Akt, and p-ERK/ERK **c** Quantitative analysis of p-Akt and p-ERK. β-Actin was used as control. Mean ± SEM, *n* = 3 independent experiments per group. The groups were compared by one-way ANOVA and followed by Dunnett’s multiple comparison tests. Differences were considered significant at **p < 0.01, ***p < 0.001. **d** Immunoblotting for ZO-1 and LC-3 in control, oxLDL and DDS treated oxLDL groups. Rapamycin was added in these groups respectively. **e** Quantitative analysis of ZO-1 and LC-3. β-Actin was used as control. Mean ± SEM, *n* = 3 independent experiments per group. **f** Immunoblotting for ZO-1 and LC-3 in control, oxLDL and DDS treated oxLDL groups. CQ was added in these groups, respectively. **g** Quantitative analysis of ZO-1 and LC-3. β-Actin was used as control. Mean ± SEM, *n* = 3 independent experiments per group. The groups were compared by two-way ANOVA, followed by Tukey’s multiple comparison tests and Sidak’s multiple comparison test. Differences were considered significant at *p < 0.05, **p < 0.01, ***p < 0.001

Next, to assess the relationship between DDS protecting tight junctions and DDS activating autophagy and lysosome function, we showed that oxLDL led to ZO-1 disruption and colocalization with LC-3 compared with the control group. DDS downregulated the colocalization rate between ZO-1 and LC-3, and recovered ZO-1 disruption (Fig. 6a). This result suggested autophagy and lysosome indeed participated in ZO-1 degradation during oxLDL treatment.

The phosphorylation of Akt (protein kinase B, PKB) and ERK1/2 (extracellular regulated protein kinases 1/2), which are involved in autophagy-related signaling pathway, was detected in the following studies. We found that oxLDL upregulated the level of p-Akt and p-ERK1/2 compared with the control (Fig. 6b, c). Although DDS decreased p-Akt and p-ERK1/2 level compared with oxLDL treatment group (Fig. 6b, c). These results indicated that DDS promoted autophagy through suppressing phosphorylation of Akt and ERK1/2 in our study. Then, we used rapamycin (100 nM, 4 h) to induce autophagy by inhibiting mTOR (mammalian target of rapamycin complex) activity. Interestingly, when cells were treated with rapamycin, rapamycin mildly increased ZO-1 in the oxLDL group (Fig. 6d, e), which suggested that ZO-1 could also be protected by activating autophagy via rapamycin during oxLDL treatment. This finding indicated that the vital role of DDS protecting tight junction proteins was activating autophagy. We also used CQ (25 μM, 4 h) to inhibit autophagosome–lysosome fusion and found that the level of ZO-1 was unchanged in the presence or absence of oxLDL (Fig. 6f, g). This result indicated that the lysosome function (downstream of autophagy) played a key role in protecting tight junctions in our models.

**Fig. 7:**
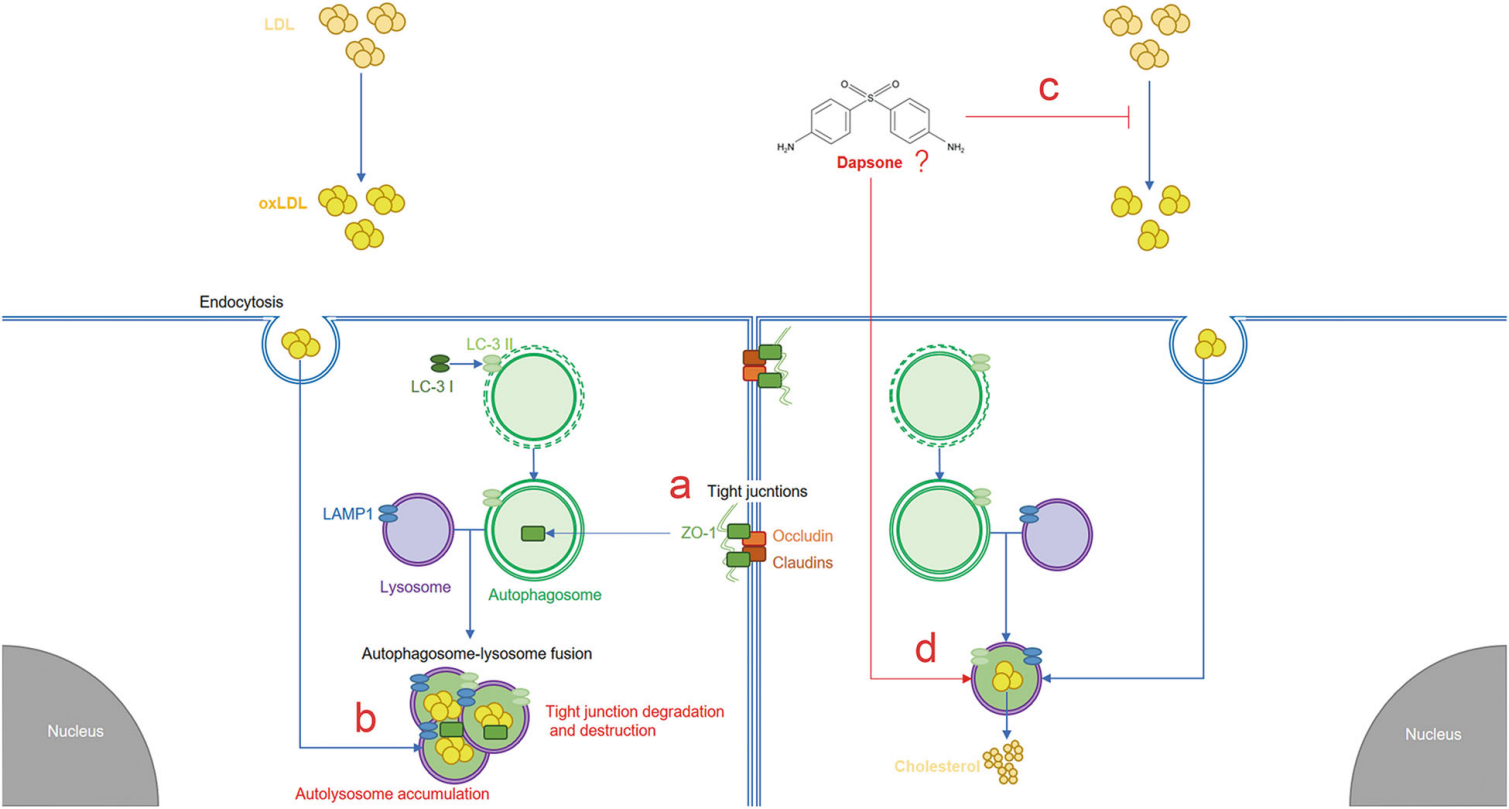
Summary scheme outlines the proposed pathway. **a** DDS protects brain microvascular integrity via protecting tight junctions. **b** oxLDL induces autolysosome accumulation, and oxLDL leads to tight junction degradation and destruction. **c** DDS decreases oxLDL level through its anti-oxidative effect. **d** DDS activating autophagy and reducing lysosome accumulation. In summary, DDS protects tight junctions via activating autophagy and reducing lysosome accumulation in oxLDL-induced brain microvascular impairment model in vivo and in vitro

Taking all these results together, we could draw the conclusion that DDS alleviated the oxLDL-induced tight junction abnormal degradation by activating autophagy through reducing lysosome accumulation (Fig. 7).

## Discussion

In the previous study, mice with HFD were established for an animal model of hyperlipidemia. In previous publications, the brain microvascular injury could be measured by the integrity degree of brain microvessels and quantified by the brain microvascular permeability of TMR-dextran (MW 40,000) from blood to brain parenchyma in a new live optical study^40^. In our study, we observed the impairment of BBB induced by HFD using this new technology: multiphoton confocal microscope in vivo for the first time. To assess the vascular protective effect of DDS, mice were given drinking water with 5 mg/kg DDS during HFD, and we used the extravasation of dye into brain parenchyma, which was injected via tail vein. We found that DDS significantly attenuated the leakage of brain microvessels during this process. Although DDS was previously proved to have neuroprotective effects on acute inflammation and ischemic stroke, our results discovered a new endothelial protective role of DDS in a lipids metabolism disorder and chronic inflammation mice model. Through studying body weight, the level of serum glucose, TC, TG and oxLDL, we found in the following experiments that DDS reversed the level of oxLDL in HFD mice. Oxidized cholesterol has damaging effects on the BBB integrity and function^41,42^. We also used in vitro results to confirm our previous findings. Cu^2+^ salts are used in the classic protocol for oxidizing LDL, which had revealed many details of oxLDL formation^43–47^. Because the macromolecule, LDL composed of a very large protein portion core, approximately 550 kD of which is surrounded by a nonpolar lipid portion^48^; this structural complexity of LDL makes its structure still fail to elucidate. CD spectroscopy allows us to analyze both the overall secondary structure and the asymmetric environment of the prosthetic heme group of LDL in vitro^49^. These in vivo and in vitro data revealed that DDS attenuated leakage of brain microvessels via reducing the level of oxLDL.

Lysosomes are organelles that are found in most eukaryotic cells. Lysosomes contain acidic enzymes: proteases, lipases and nucleases, which can degrade practically all kinds of macromolecules in vivo^50,51^. Lysosomes receive and degrade macromolecules through fusing with endosomes, autophagosomes and phagosomes. The degradation products: protein, lipid and carbohydrate can be reused by cells for bioenergetic substrates recycling and organelles turnover. Lysosome associated membrane protein 1 (LAMP1) and LAMP2 comprise 50% of all lysosomal membrane proteins, and maintain lysosomal membrane integrity^52^. LAMP1 is widely considered a lysosomal-specific marker^53,54^. The LAMPs were also found on late endosomes and cell membranes, which are known to be closely related with endosome–lysosome fusion, autophagosome–lysosome fusion and membrane repair. In cholesterol metabolism, LAMPs were found to be involved in lysosomal cholesterol export^55^. Some previous studies had reported that autophagosome–lysosome fusion was dysregulated in LAMP1 and LAMP2-deficient cells, but the underlying mechanism still remains unclear. We also found LAMP2 changed in our proteomics analysis (shown in Fig S2B).

Brain microvascular integrity is maintained by tight junction proteins^33^. Endothelial function is closely associated with tight junctions, and ZO-1 contributes to the configuration of tight junctions^33^. It is well known that endothelial cells will dysfunction when exposed to oxLDL^56^. Our results suggest that DDS increases tight junction proteins: ZO-1, occludin and claudin-5. Intriguingly, reduced levels of tight junction proteins might be connected with the degradation system^35^. The disorder of tight junctions may be associated with the autophagy. And, HFD and oxidized cholesterol may disturbed lysosome^57^. Consistent with our prediction, DDS increased the level of autophagosome membrane protein LC-3. Treated with oxLDL, larger size lysosomes increased and were full of rough granules observed by TEM. LC-3 colocalized with accumulated lysosomes with diameters of >1.5 μm, which confirmed that oxLDL induced dysfunction of autophagy and lysosome accumulation. DDS treatment reduced lysosome accumulation and increased LC-3 colocalization with lysosomes with diameters of <1.5 μm. It also restored p62 to normal level, which indicated that DDS accelerated autophagy function to normal level. LAMPs oligomerization associated with autophagy^58^ and cholesterol levels may influence LAMP protein conformation. OxLDL also induced LAMP1 colocalization with lysosome accumulation, which is consistent with previous study. Colocalization of LAMP1 with lysosomes smaller than 1.5 μm is observed to enhance after DDS treatment. However, total colocalization of LAMP1 with lysosomes did not increase after DDS treatment (Supplementary Fig. 3). In this result, lysotracker was used to detect lysosomes (a florescent cell-permeable peptide that accumulates selectively in acidic organelles such as lysosomes^59^). Lysotracker showed no difference between control and DDS treated oxLDL groups, but LAMP1 distinguished activated lysosomes in oxLDL group and DDS-treated oxLDL groups from control. This result suggested that although lysotracker could not differentiate non-activated lysosomes from activated ones, lysotracker can measure the dysfunction and accumulation of lysosomes. Because the LAMPs are related with endosome–lysosome fusion, autophagosome–lysosome fusion and membrane repairing, LAMP1 colocalization with accumulated lysosomes indicated that lysosome accumulation might induce an abnormal degradation of tight junctions (Supplementary Fig. 3). These data also explained the destruction of tight junctions on cytomembrane^60^, and was confirmed by ZO-1 colocalization with LC-3 in the experiment (Fig. 6a). To identify the mechanism of the previous phenomenon, we used mTOR inhibitor—rapamycin, which enhanced autophagy, as well as autophagosome–lysosome fusion inhibitor—CQ. There was an interesting result in Fig. 6e: the last group with oxLDL + DDS + RAPA in LC-3 II had relatively lower protein level compared with the oxLDL + RAPA or the oxLDL + DDS group. As a mTOR inhibitor, rapamycin only enhanced autophagy signaling pathway, but not influenced the downstream of autophagosome–lysosome fusion. This meant that rapamycin treatment only accelerated LC-3 lipidation and autophagy bubble formation, but not promoted autophagy flux. In our experimental design, rapamycin was only added in medium at the first 4 h (because its cytotoxicity for the long-time treatment), then oxLDL and DDS was treated continuously for the following 20 h. Considering our previous experiments, our checkpoint was defined at 24 h after drug treatment. Thus, there was a transient acceleration of LC-3 I formed to LC-3 II after rapamycin addition. Additionally, in oxLDL + RAPA group, owing to oxLDL treatment induced lysosome accumulation, blocked autophagy flux and decelerated LC-3 recycling, which made LC-3 II increasing in cytoplasm. However, DDS has more effects on reversing lysosome accumulation than facilitating autophagy. In oxLDL + DDS + RAPA group, when adding rapamycin, LC-3 II may increase at the first 4 h. Owing to rapamycin activating autophagy and DDS decreasing lysosome accumulation, the two factors consistently accelerated autophagy flux and LC-3 recycling. So, LC-3 II level was lower in oxLDL + DDS + RAPA group than in oxLDL + RAPA group or oxLDL + DDS group. According to our explanation, we found that the LC-3 II level in RAPA group was extremely high. We suggested that the baseline level of autophagy in control group was very low, rapamycin addition stimulated autophagy to a high level. There were little substrates for degradation in RAPA group than other oxLDL treatment groups, which made the cell not need to consume energy for degradation. Thus, autophagy was continuously activated with high LC-3 II level. We found that DDS protected tight junctions in an autophagy-dependent manner and lysosome played important roles in this process. OxLDL treatment influenced lipid metabolism, and affected Akt and ERK, mTOR, and autophagy-related signaling pathways in our study^61–64^. Here, we demonstrated for the first time that DDS protected brain microvascular integrity through linking tight junctions protection with the degradation system.

In previous studies, DDS has been proved to have neuroprotective effects on acute inflammation and ischemic stroke. Additionally, DDS extended lifespan in the *Caenorhabditis elegans* and Alzheimer’s disease patients. Currently studies reported that the environmental risk factors were considered to influence the occurrence and development of metabolic diseases. Previous studies have been shown that the western diets were related to our organism aging and metabolic syndromes. Whereas, the underlying mechanism between environmental factor such as HFD and metabolic syndrome had yet to be elucidated. Our previous study found that DDS has anti-inflammation effect during acute inflammation induced BBB injury. Thus, the anti-oxidative effect of DDS in the field of vascular protection and its mechanism were barely studied. And whether DDS regulating autophagy–lysosome function in the cerebral microvessel and its underlying effects was still unknown. In our investigation, we first demonstrated that DDS had a protective role on brain microvascular integrity under lipids metabolism disorder and chronic inflammation conditions. We linked DDS protecting tight junctions with activating autophagy to normal level, and we also connected DDS protecting tight junctions with reducing lysosome accumulation. DDS perhaps plays an anti-oxidative role on oxLDL, which explains the protective effect on tight junctions. Additionally, DDS has a protective role on decreasing lysosome accumulation and recovering lysosome function under abnormal degradation conditions. (Fig. 7). In summary, DDS may be used as an anti-oxidation drug to prevent cerebral microvascular integrity. Our findings suggested the significance of DDS in protecting brain microvessels under lipid metabolic disorders, which revealed a novel potential therapeutic strategy in brain microvascular related disease.

## Materials and methods

### Study approval

#### Human plasma sample

Fresh plasma was separated with centrifugation from peripheral blood obtained from healthy donors. The study protocol was approved by the local ethics committee.

#### Animals and treatment

In all, 12–16 week old male C57BL/6J mice weighing 25–30 g were fed at the Animal Center of the Peking University Health Science Center, Beijing, China. Before the experiments, mice were allowed to acclimatize for 1 week in their animal quarters with air conditioning and an automatically controlled photoperiod of 12 h of light daily.

All procedures were performed according to the National Institutes of Health Guidelines for the Use of Laboratory Animals and approved by the Institutional Animal Care and Use Committee of the Peking University Health Science Center, Beijing, China.

#### High-fat diet

Mice were randomly divided into (1) control group (normal diet), (2) HFD group, and (3) HFD with dose-dependent DDS treatment group. The normal standard diet contained 4.5% fat, 20% protein, and 52% complex carbohydrates, with a total energy of 3.29 kcal/g. The composition of the HFD included 15.7% fat, 17.6% protein, and 45.7% complex carbohydrates, with a total energy of 3.95 kcal/g.

#### Drug administration

DDS was purchased from Sigma–Aldrich (Sigma–Aldrich, St. Louis, MO, USA). DDS was dissolved in absolute ethyl alcohol containing 10% PEG-300. The mice were exposed daily to 5 mg/kg DDS of body weight by drinking water. Mice were administered an HFD and DDS for 8 weeks. Control animals received 0.5% ethanol and 0.05% PEG-300. The tap water with DDS was refreshed every 2 days. The amounts of water and food consumed and body weights were measured. At the end of the experiments, animals were sacrificed. Blood samples were collected and centrifuged to obtain serum. The brains were removed, weighed, and collected.

#### Evans blue assay

Evans blue assay was carried out as previously described^65^. Mice were deeply anesthetized with pentobarbital sodium (1%), and Evans blue dye (30 mg/ml in 0.9% saline) was injected (4 ml/kg) into the tail vein and allowed to circulate for 1 h. The mice were perfused with 20 ml of phosphate-buffered saline (PBS). The brains were homogenized in 2 ml of 0.1 M PBS. After centrifugation at 1000 *g* for 5 min at 4 °C, 0.7 ml of the supernatant was added to 0.7 ml of 100% (w/v) trichloroacetic acid. The mixture was incubated at 4 °C for 18 h and then centrifuged at 1000 *g* for 30 min at 4 °C. Evans blue concentration in the supernatant was detected at 610 nm using a spectrophotometer. The results are presented as milligrams of Evans blue by comparing it with a standard solution.

#### Multiphoton laser confocal fluorescence microscopy in vivo analysis

In vivo two-photon imaging was performed as previously described^25,66,67^. Mice were anesthetized with 40 mg/kg pentobarbital sodium and the cranium was firmly secured in a stereotaxic frame. A square cranial window was opened with a high-speed drill. TMR-conjugated dextran (40 kDa, 0.1 ml of 10 mg/ml, Invitrogen, Carlsbad, CA) was injected via the tail vein. At the end of the experiment, mice were killed by decapitation and brains were harvested, frozen and conserved at 80 °C until use. In vivo images were acquired using a two-photon microscope (Leica TCS SP5 MP, Chicago, IL) with 850 nm excitation and 20/1.0 water immersion objective, 2 mm working distance. Once the area of interest was defined, 200 μm-thick stacks in the Z-axis (5 μm steps) were obtained with the Leica ASF software. The relative fluorescence intensity across the cross-section of vessels was analyzed with ImageJ (NIH) software^25,67,68^.

#### Isolation of brain capillaries

Brain capillaries were isolated using dextran gradient centrifugation as described^25,66,67^. The cortex and hippocampus were carefully dissected and the meninges were removed in ice-cold PBS containing 2% fetal bovine serum (FBS). The brain was homogenized and dextran (70 kDa, Pharmacia) was added to a concentration of 16%. The samples were then centrifuged at 6000 *g* for 15 min. The capillary pellet located at the bottom of the tubes was collected and sequentially filtered through a 100 and 45 μm cell strainer. The capillaries remaining on top of the 45 μm cell strainer were collected in PBS.

#### Western blot analysis

Isolated brain capillaries or cultured cells after washing with PBS were lysed in RIPA buffer, and the lysates were collected. The protein concentration of each lysates was determined using a BCA kit (Pierce, USA). Western blot analysis was carried out as previously described^25,67^. Extracts (30 μg of protein) were subjected to electrophoresis, and separated proteins were transferred onto PVDF (polyvinylidene fluoride) membranes, which were then immunostained with the following primary antibodies against ZO-1 (1:500, 33-9100, Invitrogen, Carlsbad, CA), occludin (1:2000, 33-1500, Invitrogen, Carlsbad, CA), claudin-5 (1:1000, ab15106-500, Abcam, San Diego, CA), LAMP1 (1:1000, ab24170, Abcam), p62 (1:2000, PM045, Medical & Biological Laboratories, Japan), LC-3 (1:2000, L7543, Sigma–Aldrich, St. Louis, MO, USA), Akt (1:500, 9272, Cell Signaling Technology, USA), p-Akt(Thr 308) (1:500, 9275, Cell Signaling Technology), ERK1/2 (1:500, 9102, Cell Signaling Technology), p-ERK1/2 (1:500, 4377, Cell Signaling Technology), and β-actin (1:5000, A1978, Sigma–Aldrich). The membranes were incubated with peroxidase-conjugated secondary antibodies, and immunoreactive bands were visualized with an ECL system.

#### Measurement of serum LDL oxidation, glucose, TC, and TG

We used a serum separator tube (premixed with EDTA) and allowed samples to clot for 2 h at room temperature or overnight at 4 °C, before centrifugation for 15 min at approximately 2000 *g*. Levels of serum total oxLDL were measured by an oxLDL ELISA kit. Levels of serum glucose were measured by a glucose assay kit. Levels of serum TC were measured by a cholesterol assay kit. Levels of serum TG were measured by TG assay kit.

#### Isolation of LDL

LDL was isolated by ultracentrifugation as described previously^69^. LDL were dialyzed against four changes of PBS (pH = 7.4) containing 1 mM EDTA and 100 μM diethylenetriamine pentaacetic acid (DTPA) (Sigma–Aldrich, St. Louis, MO, USA), sterilized with a 0.22 μm filter, stored away from light at 4 °C and used within 2 months.

#### Cell culture

HBMECs were purchased from Cell Systems Corporation (USA), and cultured in endothelial cell medium (ECM; Science, USA), containing 5% FBS, 1% endothelial cell growth supplements and 1% penicillin/streptomycin solution in a humidified atmosphere (5% CO_2_) at 37 °C. Cultured medium was changed every 2 days. Although cells were growing to 80% confluence, PBS was added into the medium in control group, 200 μg/ml oxLDL was added in experimental group; and different doses of DDS were added together with oxLDL in drug treatment group. Cells were washed by PBS, collected and prepared for follow-up experiments 24 h later.

#### Drug administration

DDS was dissolved in dimethyl sulfoxide (Sigma–Aldrich) and diluted with saline solution before administration. The final concentration of dimethyl sulfoxide in vehicle was limited to 0.05% (v/v).

#### Transmission electron microscope

In a 100 mm Corning culture dish, 90% confluent cells were prepared for electron microscopy. After 2.5% glutaraldehyde fixation, the samples were gradiently dehydrated in acetone. Then samples were negatively stained with 2% phosphotungstic acid, placed onto a carbon-coated perforated film supported by a copper grid, and examined using a JEM-1400 plus transmission electron microscope (JEOL) at 100 kV.

#### Immunocytochemistry

For immunocytochemistry (ICC), immunofluorescent staining was performed as described previously^70,71^. Briefly, the following proteins were detected with the following primary antibodies against ZO-1 (1:100, 33-9100, Invitrogen), LAMP1 (1:500, ab24170, Abcam), p62 (1:500, PM045, Medical & Biological Laboratories), and LC-3 (1:1000, L7543, Sigma–Aldrich) for HBMECs, and secondary antibody goat IgG anti-rabbit dylight 488 (1:1000, 35553, Invitrogen), goat IgG anti-mouse dylight 594 (1:1000, 35511, Invitrogen). Goat IgG or PBS was used as negative control. Lysosome localization was detected by Lysotracker (Thermo Fisher Scientific, Waltham). Nuclear localization was counterstained with 4,6-diamidino-2-phenylindole (DAPI). Images were obtained under a laser-scanning confocal microscope (Leica TCS SP8, Chicago, IL). The percentage colocalized puncta was determined by Leica QWin Analysis Software.

#### Statistics

All experiments were performed in duplicate and repeated at least three times. At least two investigators finished and analyzed every experimental data independently. Data were expressed as mean ± SEM. The treatment groups were compared by one-way analysis of variance (ANOVA), followed by Dunnett’s multiple comparison tests, or two-way ANOVA followed by Tukey’s multiple comparison tests and Sidak’s multiple comparison test, for the control with GraphPad Prism 6. Differences were considered significant at *p < 0.05.

Experimental procedures for LDL oxidation in vitro: Cu2+ oxidation of LDL, enzymatic oxidation of LDL (MPO-LDL), MPO peroxidase activity assay and CD are described in the SI Materials and Methods.

## Electronic supplementary material

Supplemental Information

## References

[1] Libby P, Ridker PM, Hansson GK (2011). Progress and challenges in translating the biology of atherosclerosis. Nature.

[2] Wen Y, Leake DS (2007). Low density lipoprotein undergoes oxidation within lysosomes in cells. Circ. Res..

[3] Jeurissen ML (2016). Prevention of oxLDL uptake leads to decreased atherosclerosis in hematopoietic NPC1-deficient Ldlr-/- mice. Atherosclerosis.

[4] Leeuwenburgh C (1997). Mass spectrometric quantification of markers for protein oxidation by tyrosyl radical, copper, and hydroxyl radical in low density lipoprotein isolated from human atherosclerotic plaques. J. Biol. Chem..

[5] Steinberg D, Parthasarathy S, Carew TE, Khoo JC, Witztum JL (1989). Beyond cholesterol. Modifications of low-density lipoprotein that increase its atherogenicity. N. Engl. J. Med..

[6] Berliner JA (1995). Atherosclerosis: basic mechanisms. Oxidation, inflammation, and genetics. Circulation.

[7] Fraley AE, Tsimikas S (2006). Clinical applications of circulating oxidized low-density lipoprotein biomarkers in cardiovascular disease. Curr. Opin. Lipidol..

[8] Tsimikas S (2008). In vivo markers of oxidative stress and therapeutic interventions. Am. J. Cardiol..

[9] Yoshida H, Kisugi R (2010). Mechanisms of LDL oxidation. Clin. Chim. Acta.

[10] Ueno M (2011). The expression of CD36 in vessels with blood-brain barrier impairment in a stroke-prone hypertensive model. Neuropathol. Appl. Neurobiol..

[11] Theriault P, ElAli A, Rivest S (2016). High fat diet exacerbates Alzheimer’s disease-related pathology in APPswe/PS1 mice. Oncotarget.

[12] Lee SW (2003). SSeCKS regulates angiogenesis and tight junction formation in blood-brain barrier. Nat. Med..

[13] Mizushima N, Komatsu M (2011). Autophagy: renovation of cells and tissues. Cell.

[14] Levine B, Kroemer G (2008). Autophagy in the pathogenesis of disease. Cell.

[15] Farkas E, Luiten PG (2001). Cerebral microvascular pathology in aging and Alzheimer’s disease. Prog. Neurobiol..

[16] Guan J, Mathai S, Liang HP, Gunn AJ (2013). Insulin-like growth factor-1 and its derivatives: potential pharmaceutical application for treating neurological conditions. Recent. Pat. Cns. Drug. Discov..

[17] Grunwald MH, Amichai B (1996). Dapsone - the treatment of infectious and inflammatory diseases in dermatology. Int. J. Antimicrob. Agents.

[18] Cho SC (2010). DDS, 4,4′-diaminodiphenylsulfone, extends organismic lifespan. Proc. Natl. Acad. Sci. USA.

[19] Nader-Kawachi J, Gongora-Rivera F, Santos-Zambrano J, Calzada P, Rios C (2007). Neuroprotective effect of dapsone in patients with acute ischemic stroke: a pilot study. Neurol. Res..

[20] Chui DH, Tabira T, Izumi S, Koya G, Ogata J (1994). Decreased beta-amyloid and increased abnormal Tau deposition in the brain of aged patients with leprosy. Am. J. Pathol..

[21] Hill QA (2015). How does dapsone work in immune thrombocytopenia? Implications for dosing. Blood.

[22] Lan CC, Wu CS, Huang SM, Wu IH, Chen GS (2013). High-glucose environment enhanced oxidative stress and increased interleukin-8 secretion from keratinocytes: new insights into impaired diabetic wound healing. Diabetes.

[23] Zhang T (2015). Surgical stress induced depressive and anxiety like behavior are improved by dapsone via modulating NADPH oxidase level. Neurosci. Lett..

[24] Yang N (2017). Protective effect of dapsone on cognitive impairment induced by propofol involves hippocampal autophagy. Neurosci. Lett..

[25] Zhou T (2014). Blood-brain barrier dysfunction in mice induced by lipopolysaccharide is attenuated by dapsone. Biochem. Biophys. Res. Commun..

[26] Wozel G, Blasum C (2014). Dapsone in dermatology and beyond. Arch. Dermatol. Res..

[27] Spatz M, Klatzo I (1976). Pathological aspects of brain transport phenomena. Adv. Exp. Med. Biol..

[28] Lusis AJ (2000). Atherosclerosis. Nature.

[29] Tsimikas S, Miller YI (2011). Oxidative modification of lipoproteins: mechanisms, role in inflammation and potential clinical applications in cardiovascular disease. Curr. Pharm. Des..

[30] Klebanoff SJ (2005). Myeloperoxidase: friend and foe. J. Leukoc. Biol..

[31] Arnhold J, Flemmig J (2010). Human myeloperoxidase in innate and acquired immunity. Arch. Biochem. Biophys..

[32] Moguilevsky N (2004). Monoclonal antibodies against LDL progressively oxidized by myeloperoxidase react with ApoB-100 protein moiety and human atherosclerotic lesions. Biochem. Biophys. Res. Commun..

[33] Huber JD, Egleton RD, Davis TP (2001). Molecular physiology and pathophysiology of tight junctions in the blood-brain barrier. Trends Neurosci..

[34] Niu C (2016). Macrophage foam cell-derived extracellular vesicles promote vascular smooth muscle cell migration and adhesion. J. Am. Heart Assoc..

[35] Nighot PK, Hu CA, Ma TY (2015). Autophagy enhances intestinal epithelial tight junction barrier function by targeting claudin-2 protein degradation. J. Biol. Chem..

[36] Mellman I (1989). Organelles observed: lysosomes. Science.

[37] Bandyopadhyay D, Cyphersmith A, Zapata JA, Kim YJ, Payne CK (2014). Lysosome transport as a function of lysosome diameter. PLoS. One..

[38] Ohsumi Y (2014). Historical landmarks of autophagy research. Cell Res..

[39] Walkley SU, Vanier MT (2009). Secondary lipid accumulation in lysosomal disease. Biochim. Biophys. Acta.

[40] Bell RD (2010). Pericytes control key neurovascular functions and neuronal phenotype in the adult brain and during brain aging. Neuron.

[41] Dias IH, Polidori MC, Griffiths HR (2014). Hypercholesterolaemia-induced oxidative stress at the blood-brain barrier. Biochem. Soc. Trans..

[42] Gamba P (2015). Oxidized cholesterol as the driving force behind the development of Alzheimer’s disease. Front. Aging Neurosci..

[43] Esterbauer H, Striegl G, Puhl H, Rotheneder M (1989). Continuous monitoring of in vitro oxidation of human low density lipoprotein. Free Radic. Res. Commun..

[44] Kleinveld HA, Hak-Lemmers HL, Stalenhoef AF, Demacker PN (1992). Improved measurement of low-density-lipoprotein susceptibility to copper-induced oxidation: application of a short procedure for isolating low-density lipoprotein. Clin. Chem..

[45] Steinbrecher UP, Parthasarathy S, Leake DS, Witztum JL, Steinberg D (1984). Modification of low density lipoprotein by endothelial cells involves lipid peroxidation and degradation of low density lipoprotein phospholipids. Proc. Natl. Acad. Sci. USA.

[46] Lynch SM, Frei B (1995). Reduction of copper, but not iron, by human low density lipoprotein (LDL). Implications for metal ion-dependent oxidative modification of LDL. J. Biol. Chem..

[47] Ziouzenkova O, Sevanian A, Abuja PM, Ramos P, Esterbauer H (1998). Copper can promote oxidation of LDL by markedly different mechanisms. Free Radic. Biol. Med..

[48] Brown MS, Kovanen PT, Goldstein JL (1981). Regulation of plasma cholesterol by lipoprotein receptors. Science.

[49] Delporte C (2014). Impact of myeloperoxidase-LDL interactions on enzyme activity and subsequent posttranslational oxidative modifications of apoB-100. J. Lipid Res..

[50] Xu H, Ren D (2015). Lysosomal physiology. Annu. Rev. Physiol..

[51] Saftig P, Klumperman J (2009). Lysosome biogenesis and lysosomal membrane proteins: trafficking meets function. Nat. Rev. Mol. Cell Biol..

[52] Wilke S, Krausze J, Bussow K (2012). Crystal structure of the conserved domain of the DC lysosomal associated membrane protein: implications for the lysosomal glycocalyx. BMC Biol..

[53] Stenmark H (2009). Rab GTPases as coordinators of vesicle traffic. Nat. Rev. Mol. Cell Biol..

[54] Zhao Z (2015). Central role for PICALM in amyloid-beta blood-brain barrier transcytosis and clearance. Nat. Neurosci..

[55] Li J, Pfeffer SR (2016). Lysosomal membrane glycoproteins bind cholesterol and contribute to lysosomal cholesterol export. eLife.

[56] Gardner G, Banka CL, Roberts KA, Mullick AE, Rutledge JC (1999). Modified LDL-mediated increases in endothelial layer permeability are attenuated with 17 beta-estradiol. Arterioscler. Thromb. Vasc. Biol..

[57] Sano R, Reed JC (2013). ER stress-induced cell death mechanisms. Biochim. Biophys. Acta.

[58] Bandyopadhyay U, Kaushik S, Varticovski L, Cuervo AM (2008). The chaperone-mediated autophagy receptor organizes in dynamic protein complexes at the lysosomal membrane. Mol. Cell. Biol..

[59] Biggs JR (2001). The human brm protein is cleaved during apoptosis: the role of cathepsin G. Proc. Natl. Acad. Sci. USA.

[60] Luzio JP, Pryor PR, Bright NA (2007). Lysosomes: fusion and function. Nat. Rev. Mol. Cell Biol..

[61] Hundal RS (2001). Oxidized low density lipoprotein inhibits macrophage apoptosis through activation of the PI 3-kinase/PKB pathway. J. Lipid Res..

[62] Noda T, Ohsumi Y (1998). Tor, a phosphatidylinositol kinase homologue, controls autophagy in yeast. J. Biol. Chem..

[63] Zoncu R, Efeyan A, Sabatini DM (2011). mTOR: from growth signal integration to cancer, diabetes and ageing. Nat. Rev. Mol. Cell Biol..

[64] Thoreen CC (2009). An ATP-competitive mammalian target of rapamycin inhibitor reveals rapamycin-resistant functions of mTORC1. J. Biol. Chem..

[65] Qi X, Inagaki K, Sobel RA, Mochly-Rosen D (2008). Sustained pharmacological inhibition of deltaPKC protects against hypertensive encephalopathy through prevention of blood-brain barrier breakdown in rats. J. Clin. Invest..

[66] Bell RD (2012). Apolipoprotein E controls cerebrovascular integrity via cyclophilin A. Nature.

[67] Zhou T (2014). Phospholipid transfer protein (PLTP) deficiency impaired blood-brain barrier integrity by increasing cerebrovascular oxidative stress. Biochem. Biophys. Res. Commun..

[68] Ruiz-Valdepenas L (2011). Cannabidiol reduces lipopolysaccharide-induced vascular changes and inflammation in the mouse brain: an intravital microscopy study. J. Neuroinflamm..

[69] Orsoni A (2011). LDL-apheresis depletes apoE-HDL and pre-beta1-HDL in familial hypercholesterolemia: relevance to atheroprotection. J. Lipid Res..

[70] Tong Y (2015). Phospholipid transfer protein (PLTP) deficiency accelerates memory dysfunction through altering amyloid precursor protein (APP) processing in a mouse model of Alzheimer’s disease. Hum. Mol. Genet..

[71] Xian X (2009). Presynaptic defects underlying impaired learning and memory function in lipoprotein lipase-deficient mice. J. Neurosci..

