# Supplementary material for: Dapsone protects brain microvascular integrity from high-fat diet induced LDL oxidation
Source: Cell Death Dis. 2018 Jun 7;9(6):683. doi: 10.1038/s41419-018-0739-y (PMC5992187; doi:10.1038/s41419-018-0739-y)
Supplement: Supplementary file 1 — Supplemental Information [file 41419_2018_739_MOESM1_ESM.docx]

**Supplemental information**

**Supplementary materials and methods**

***LDL oxidation in vitro***

***Cu^2+^ oxidation of LDL***

The *in vitro* oxidation of LDL was performed by using a modification of the procedure previously described[^1^](#_ENREF_1). Before the start of the oxidation, the LDL (0.1 g of LDL protein per well as a final concentration) was added in every well, while the treatment groups were pre-treated with dose-dependent DDS of final concentrations 1 µM, 5 µM, 10 µM, 25 µM, 50 µM, 100 µM. Oxidation was initiated by adding a freshly prepared CuSO_4_ solution (final concentration 5 µmol/L). Blank control was performed using LDL incubated with Cu^2+^ in the absence of DDS, while positive control was performed using native LDL treatment with butylated hydroxytoluene (BHT) (Sigma–Aldrich, St. Louis, MO, USA), and negative control was performed using complete oxidative LDL, which was acquired from LDL pre-incubated with divalent copper ion for 24 h. The kinetics of LDL oxidation were determined by monitoring the change in absorbance at 234 nm at 37℃ with a multifunctional enzyme mark (Thermo Fisher Scientific, Waltham), equipped with a 96 well UV star plate (Corning, Corning), allowing for as many as 96 samples to be measured simultaneously. Absorbance was recorded every 5 min for 3 h. The change in absorbance at 234 nm, versus time, could be divided into three consecutive phases: lag, propagation, and decomposition. After this step of *in vitro* oxidation, we used an oxLDL ELISA kit (Cloud-Clone, Houston) to detect the concentration of oxLDL in every well.

***Enzymatic oxidation of LDL (MPO-LDL)***

A more intensive enzymatic oxidation process was performed as previously established[^2^](#_ENREF_2). Briefly, before the start of the oxidation, the LDL was added in every well, while the experimental groups were pre-treated with dose-dependent DDS, and the final concentration of DDS was divided into 1 µM, 5 µM, 10 µM , 50 µM, 100 µM. Blank control was performed using LDL incubated with MPO (Sigma–Aldrich, St. Louis, MO, USA) in the absence of DDS, while positive control was performed using native LDL treatment with a lipid antioxidant BHT, and negative control was performed using complete oxidative LDL, which was acquired from LDL pre-incubated with Cu^2+^ for 24 h. Briefly, MPO-LDL was generated by mixing 2 µL of 1 M HCl (final concentration: 10 mM), 10 µl of dose dependent DDS, 20 µl of MPO (final concentration: 0.3 mg/ml), 20 µl of LDL (final concentration: 0.1 mg/ml in 10 mM PBS, pH 7.4), and 10 µl of H_2_O_2_ (final concentration: 1 mM). The volume was adjusted to 200 µl with NaAc (pH 5.4). LDL was incubated with MPO, HCl and H_2_O_2_ for 2 h at 37℃ at pH 5.4. After this step of *in vitro* oxidation, we used an oxLDL ELISA kit to detect the concentration of oxLDL in every well.

***MPO peroxidase activity assay***

The residual peroxidase activity of MPO was determined by measuring its ability to oxidize tetramethylbenzidine (TMB) (Sigma–Aldrich, St. Louis, MO, USA) [^3^](#_ENREF_3). MPO (0.3 mg/ml) was dissolved in 50 mM sodium phosphate buffer, pH 7.4, and 100 µM DTPA. Residual peroxidase activity was measured by adding 20 µl LDL and 10 µl dose-dependent DDS to 150 µl of 200 mM sodium acetate buffer, pH 5.4, containing 0.01% cetrimide, 10 µl of 20 mM TMB in dimethyl formamide (DMF) (Sigma–Aldrich, St. Louis, MO, USA), which was made freshly each day and kept in the dark, and 10 µl of 10 mM hydrogen peroxide. Reactions were performed at 37℃ and started by addition of the 20 µl MPO. TMB oxidation was followed at 670 nm and initial rates were calculated over the first 60 s of the reaction.

***Circular dichroism***

Circular dichroism spectra were performed on Chirascan spectrometer equipment (Jasco, Japan). For recording far-UV spectra (200-260 nm), and near-UV visible region (260-480 nm), conditions were as follows: path length; 1 mm, spectral bandwidth; 1 nm, step size; 1 nm, scan time by nanometer; 10 s, protein concentrations; 0.3 mg/ml MPO and 0.1 mg/ml LDL. We used LDL pre-treated with 20 µM DDS (a mild concentration), as the experimental groups. To every samples, we added Cu^2+^ as oxidant for 2 h at 37℃ before the experiments. Positive control was performed using native LDL in the absence of Cu^2+^. All CD measurements were performed in 5 mM PBS (pH 7.4) at 25℃^[4](#_ENREF_4)^. Each spectrum was automatically corrected with the baseline to remove birefringence of the sample chamber.

***Statistics***

To statistically analyze the UV absorbance curve among the treatment groups, we employed the Boltzman sigmoidal nonlinear regression model; the computational formula was Y=Bottom+(Top-Bottom)/[1+exp((V50-X)/Slope)], lag time=X-2/Slope, analyzed by one-way ANOVA followed by Dunnett`s multiple comparison tests. To statistically analyze the residual peroxidase activity of MPO among the treatment MPO-LDL groups, we employed the log (inhibitor) vs. response -- Variable slope model in GraphPad Prism 6, analyzed by one-way ANOVA followed by Dunnett`s multiple comparison tests. Differences were considered significant at *p＜0.05.

**Supplementary figures and figure legends**

***
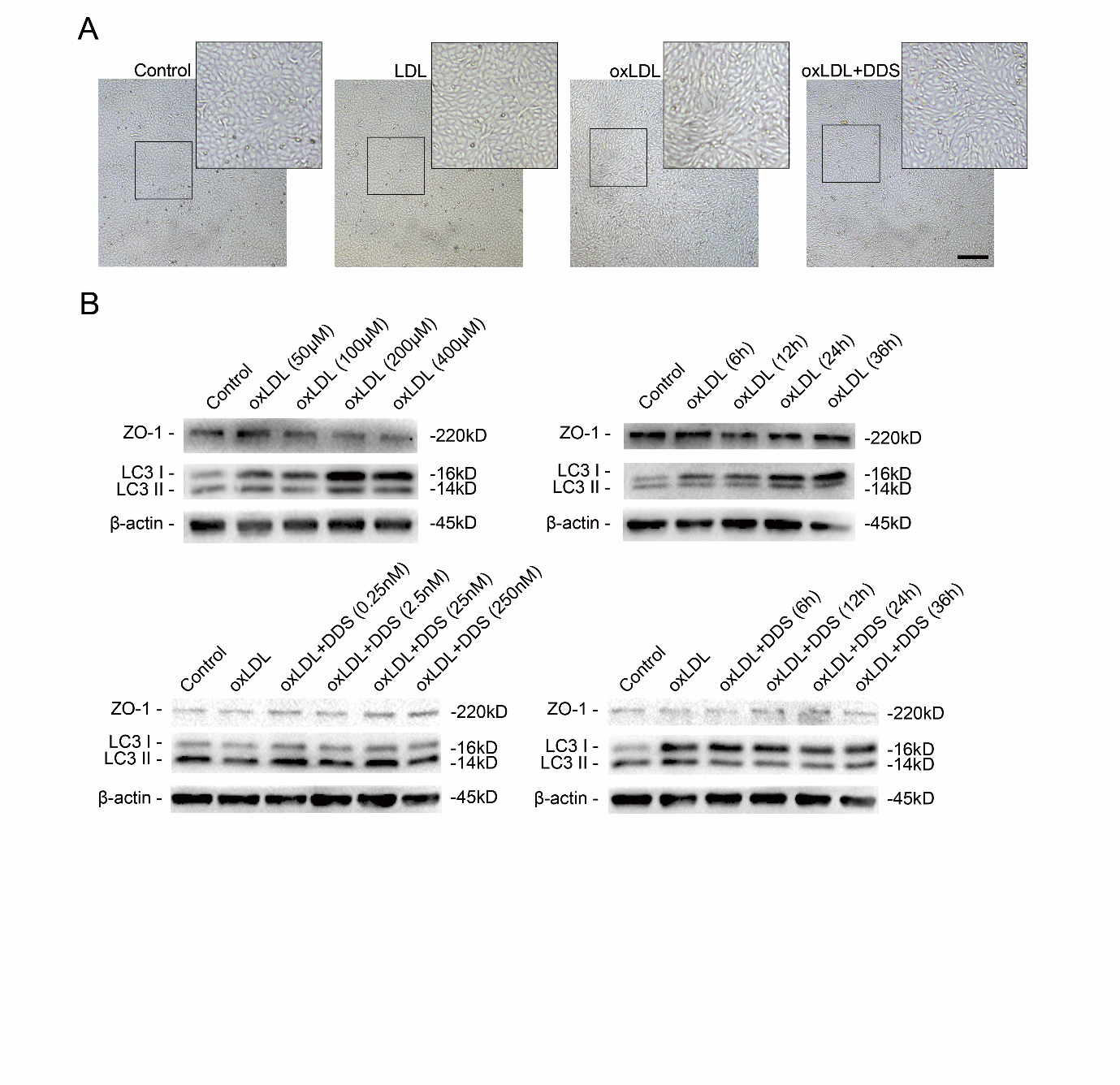
Supplementary figure. 1 Dose-dependent manner of DDS protects tight junctions and regulates autophagy.***

(A) Cell morphology of HBMECs treated with PBS (control), LDL, oxLDL and oxLDL with DDS. (B) Immunoblotting for tight junction protein (ZO-1) and autophagy protein (LC-3) in control, oxLDL (dose-dependent manner: 50 µg/ml, 100 µg/ml, 200 µg/ml, 400 µg/ml; time-dependent manner: 6 h, 12 h, 24 h, 36h) and DDS treated oxLDL (dose-dependent manner: 0.25nM, 2.5nM, 25nM, 250 nM; time-dependent manner: 6 h, 12 h, 24 h, 36h) groups. n=3 independent experiments per group. Scale bar, 100μm.


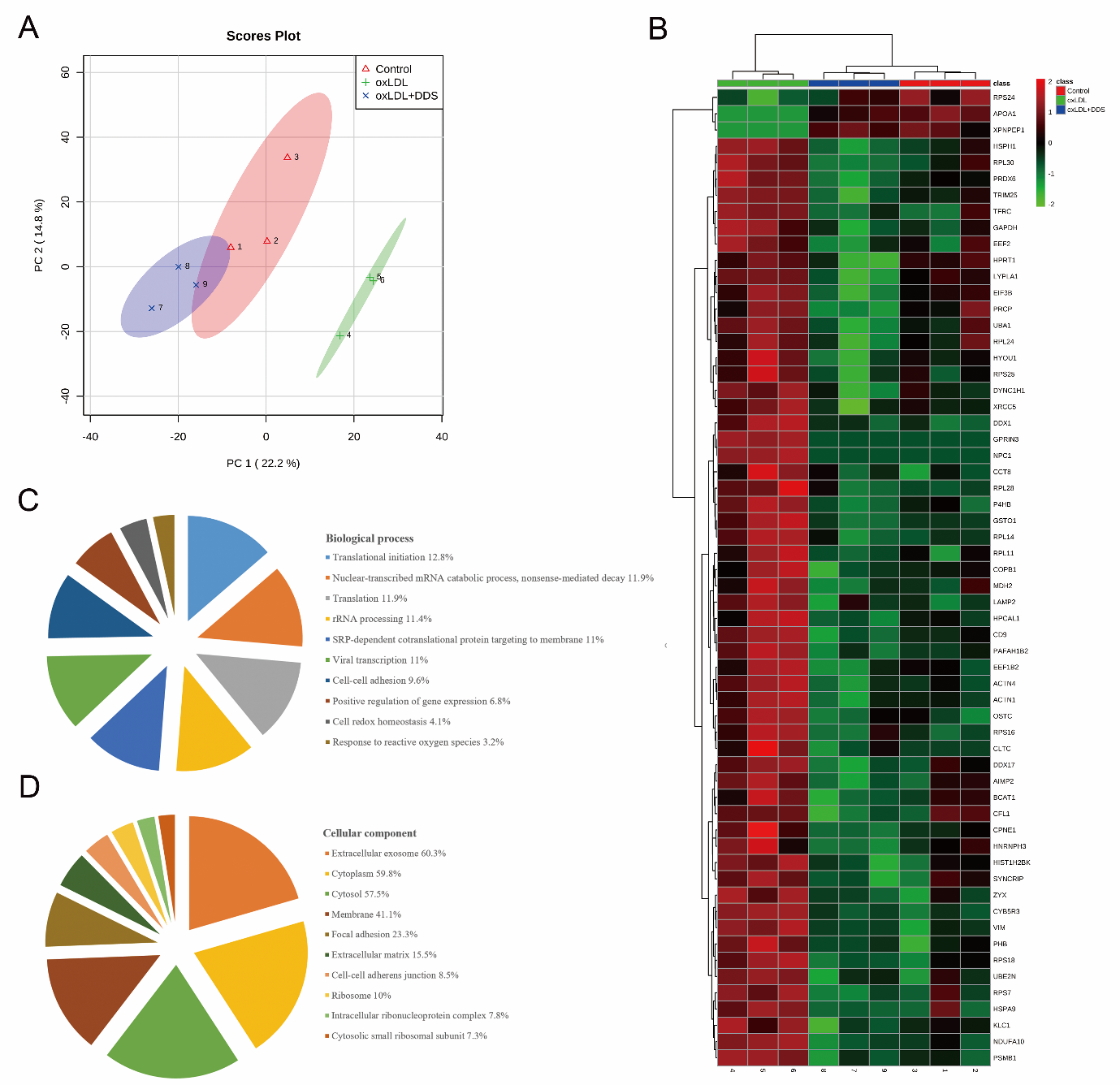
***Supplementary figure. 3 Proteomic analysis of DDS treatment in endothelial cells treated with ox-LDL.***

(A) Principal Component Analysis. (B) Heatmap from proteomics analysis. (C and D) Gene Ontology (GO) enrichment in biological processes and cellular components, respectively.

***
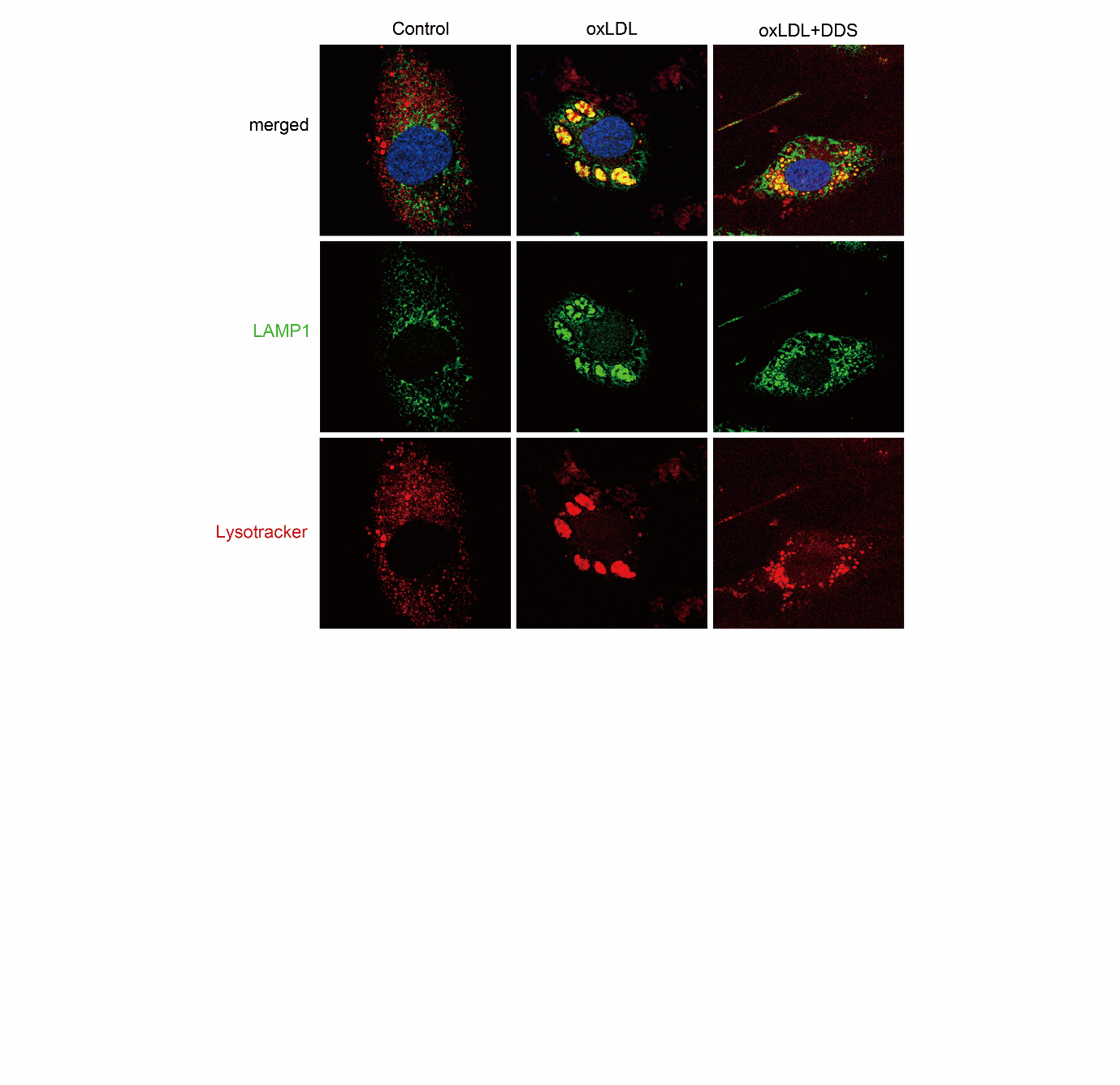
Supplementary figure. 3 LAMP1 and Lysotracker reveals lysosome accumulation consistently.***

(A) LAMP1 (Green) co-localization with lysosomes (Red, stained by Lysotracker) in control, oxLDL, and DDS treated oxLDL groups. Scale bar, 50μm.

**Reference**

1. Kleinveld HA, Hak-Lemmers HL, Stalenhoef AF, Demacker PN. Improved measurement of low-density-lipoprotein susceptibility to copper-induced oxidation: application of a short procedure for isolating low-density lipoprotein. *Clinical chemistry* 1992, **38**(10)**:** 2066-2072.

2. Moguilevsky N, Zouaoui Boudjeltia K, Babar S, Delree P, Legssyer I, Carpentier Y*, et al.* Monoclonal antibodies against LDL progressively oxidized by myeloperoxidase react with ApoB-100 protein moiety and human atherosclerotic lesions. *Biochemical and biophysical research communications* 2004, **323**(4)**:** 1223-1228.

3. Paumann-Page M, Furtmuller PG, Hofbauer S, Paton LN, Obinger C, Kettle AJ. Inactivation of human myeloperoxidase by hydrogen peroxide. *Archives of biochemistry and biophysics* 2013, **539**(1)**:** 51-62.

4. Furtmuller PG, Zederbauer M, Jantschko W, Helm J, Bogner M, Jakopitsch C*, et al.* Active site structure and catalytic mechanisms of human peroxidases. *Archives of biochemistry and biophysics* 2006, **445**(2)**:** 199-213.
